# 2-{(*E*)-1-[2-(2-Nitro­phen­yl)hydrazin-1-yl­idene]eth­yl}benzene-1,3-diol mono­hydrate

**DOI:** 10.1107/S1600536812006241

**Published:** 2012-02-17

**Authors:** R. Alan Howie, James L. Wardell, Solange M. S. V. Wardell, Edward R. T. Tiekink

**Affiliations:** a Department of Chemistry, University of Aberdeen, Meston Walk, Old Aberdeen AB24 3UE, Scotland; b Centro de Desenvolvimento Tecnológico em Saúde (CDTS), Fundação Oswaldo Cruz (FIOCRUZ), Casa Amarela, Campus de Manguinhos, Avenida Brasil 4365, 21040-900 Rio de Janeiro, RJ, Brazil; c CHEMSOL, 1 Harcourt Road, Aberdeen AB15 5NY, Scotland; d Department of Chemistry, University of Malaya, 50603 Kuala Lumpur, Malaysia

## Abstract

The hydrazone mol­ecule in title monohydrate, C_14_H_13_N_3_O_4_·H_2_O, is almost coplanar, the dihedral angle between the terminal benzene rings being 3.22 (15)°; the nitro group is coplanar with the benzene ring to which it is bonded [O—N—C—C = −2.8 (4)°]. The hy­droxy group forms an intra­molecular hydrogen bond with the imine N atom, and the conformation about the imine bond [1.305 (3) Å] is *E*. In the crystal, supra­molecular layers in the (203) plane are connected into a double layer *via* water–nitro O—H⋯O hydrogen bonds, along with π–π inter­actions [ring centroid–centroid distance = 3.7859 (19) Å].

## Related literature

For background on the influence of substituents upon the supra­molecular structures of hydrazones, see: Glidewell *et al.* (2004 ▶); Ferguson *et al.* (2005 ▶); Baddeley *et al.* (2009 ▶).
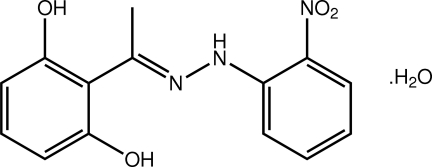


## Experimental

### 

#### Crystal data

C_14_H_13_N_3_O_4_·H_2_O
*M* *_r_* = 305.29
Monoclinic, 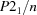

*a* = 7.6448 (6) Å
*b* = 21.405 (2) Å
*c* = 8.5755 (7) Å
β = 106.976 (5)°
*V* = 1342.1 (2) Å^3^
*Z* = 4
Mo *K*α radiation
μ = 0.12 mm^−1^
*T* = 120 K
0.45 × 0.25 × 0.02 mm

#### Data collection

Bruker–Nonius Roper CCD camera on κ-goniostat diffractometer
Absorption correction: multi-scan (*SADABS*; Sheldrick, 2007 ▶) *T*_min_ = 0.776, *T*_max_ = 0.998
16295 measured reflections
3068 independent reflections
1492 reflections with *I* > 2σ(*I*)
*R*_int_ = 0.110

#### Refinement

*R*[*F* ^2^ > 2σ(*F* ^2^)] = 0.066
*wR*(*F* ^2^) = 0.194
*S* = 1.01
3068 reflections
221 parameters
6 restraints
H atoms treated by a mixture of independent and constrained refinement
Δρ_max_ = 0.37 e Å^−3^
Δρ_min_ = −0.30 e Å^−3^

### 

Data collection: *COLLECT* (Hooft, 1998 ▶); cell refinement: *DENZO* (Otwinowski & Minor, 1997 ▶) and *COLLECT*; data reduction: *DENZO* and *COLLECT*; program(s) used to solve structure: *SHELXS97* (Sheldrick, 2008 ▶); program(s) used to refine structure: *SHELXL97* (Sheldrick, 2008 ▶); molecular graphics: *ORTEP-3* (Farrugia, 1997 ▶) and *DIAMOND* (Brandenburg, 2006 ▶); software used to prepare material for publication: *publCIF* (Westrip, 2010 ▶).

## Supplementary Material

Crystal structure: contains datablock(s) global, I. DOI: 10.1107/S1600536812006241/pv2513sup1.cif

Structure factors: contains datablock(s) I. DOI: 10.1107/S1600536812006241/pv2513Isup2.hkl

Supplementary material file. DOI: 10.1107/S1600536812006241/pv2513Isup3.cml

Additional supplementary materials:  crystallographic information; 3D view; checkCIF report

## Figures and Tables

**Table 1:** Hydrogen-bond geometry (Å, °)

*D*—H⋯*A*	*D*—H	H⋯*A*	*D*⋯*A*	*D*—H⋯*A*
O1—H1O⋯N1	0.85 (2)	1.69 (2)	2.489 (3)	157 (4)
N2—H2N⋯O3	0.88 (2)	1.93 (2)	2.602 (3)	132 (2)
O2—H2O⋯O1w^i^	0.84 (3)	1.90 (3)	2.742 (3)	174 (2)
O1*W*—H1*W*⋯O1^ii^	0.84 (2)	2.08 (3)	2.910 (3)	169 (3)
O1*W*—H2*W*⋯O4^iii^	0.85 (3)	2.50 (3)	3.256 (3)	150 (3)
C11—H11⋯O3^iv^	0.95	2.52	3.447 (4)	166

Symmetry codes: (i) 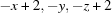; (ii) 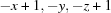; (iii) 
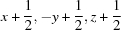
; (iv) 
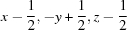
.

**Fig. 1.**
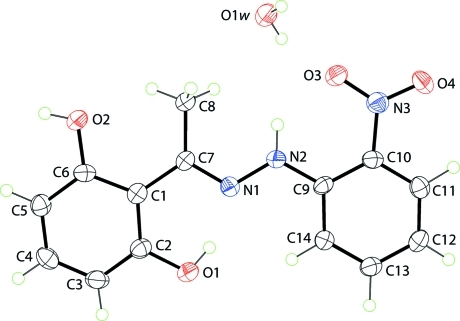
The molecular structures of the constituents of (I) showing the atom-labelling scheme and displacement ellipsoids at the 50% probability level.

**Fig. 2.**
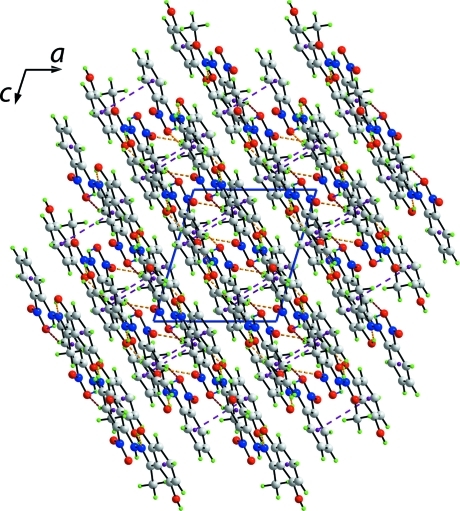
A view in projection down the b axis of the packing of supramolecular double layers in (I). The O—H···O (orange), O—H···N (orange), N—H···O (blue), C—H···O (brown) and π–π (purple) interactions are shown as dashed lines.

## supplementary crystallographic information

### Comment

The crystal structure of the title compound (I), has been determined in connection with on-going investigations into the structural chemistry of hydrazones, focusing in particular upon the influence of substituents upon their supramolecular structures, with a special emphasis on derivatives having potential biological activities. These studies have included investigations on substituted phenylhydrazines with substituted benzaldehydes (Glidewell *et al.*, 2004; Ferguson *et al.*, 2005) and 2-hydroxyacetophenone (Baddeley *et al.*, 2009).

In (I) (Fig. 1), the dihedral angle between the benzene rings is 3.22 (15)°, indicating an approximately planar molecule. The nitro group is co-planar with the benzene ring to which it is bonded as seen in the value of the O3—N3—C10—C9 torsion angle of -2.8 (4)°. The hydroxy group forms an intramolecular hydrogen bond with the imine-N1 atom, Table 1. The configuration about the N1═C7 imine bond [1.305 (3) Å] is *E*.

With the exception of the O1w—H2w···O4^iii^ hydrogen bond, all the interactions listed in Table 1 combine to form supramolecular layers parallel to (203). These are connected into double layers *via* the O1w—H2w···O4^iii^ hydrogen bonds and π–π interactions [ring centroid···centroid distance = 3.7859 (19) Å, angle between rings = 3.22 (15)° for *i*: 1 - *x*, -*y*, 1 - *z*]. Layers stack without specific interactions between them (Fig. 2).

### Experimental

A solution of 2-nitrophenylhydrazine and 2,6-dihydroxyacetophenone (2 mmol each) in ethanol (20 ml) was refluxed for 1 h, rotary evaporated and the residue recrystallized from methanol, *m*.p. 452–454 K.

### Refinement

The *C*-bound H atoms were geometrically placed (C—H = 0.95–0.98 Å) and refined as riding with *U*_iso_(H) = 1.2–1.5*U*_eq_(C). The *O*- and *N*-bound H atoms were located from a difference map and refined with the distance restraints O—H = 0.84±0.01 and N—H = 0.88±0.01 Å, and with *U*_iso_(H) = *zU*_eq_(carrier atom); *z* = 1.5 for O and *z* = 1.2 for N.

### Crystal data

**Table:**

C_14_H_13_N_3_O_4_·H_2_O	*F*(000) = 640
*M**_r_* = 305.29	*D*_x_ = 1.511 Mg m^−^^3^
Monoclinic, *P*2_1_/*n*	Mo *K*α radiation, λ = 0.71073 Å
Hall symbol: -P 2yn	Cell parameters from 19763 reflections
*a* = 7.6448 (6) Å	θ = 2.9–27.5°
*b* = 21.405 (2) Å	µ = 0.12 mm^−^^1^
*c* = 8.5755 (7) Å	*T* = 120 K
β = 106.976 (5)°	Plate, brown
*V* = 1342.1 (2) Å^3^	0.45 × 0.25 × 0.02 mm
*Z* = 4	

### Data collection

**Table:**

Bruker-Nonius Roper CCD camera on κ-goniostat diffractometer	3068 independent reflections
Radiation source: Bruker–Nonius FR591 rotating anode	1492 reflections with *I* > 2σ(*I*)
Graphite monochromator	*R*_int_ = 0.110
Detector resolution: 9.091 pixels mm^-1^	θ_max_ = 27.5°, θ_min_ = 2.9°
φ & ω scans	*h* = −9→9
Absorption correction: multi-scan (*SADABS*; Sheldrick, 2007)	*k* = −27→27
*T*_min_ = 0.776, *T*_max_ = 0.998	*l* = −11→11
16295 measured reflections	

### Refinement

**Table:**

Refinement on *F*^2^	Primary atom site location: structure-invariant direct methods
Least-squares matrix: full	Secondary atom site location: difference Fourier map
*R*[*F*^2^ > 2σ(*F*^2^)] = 0.066	Hydrogen site location: inferred from neighbouring sites
*wR*(*F*^2^) = 0.194	H atoms treated by a mixture of independent and constrained refinement
*S* = 1.01	*w* = 1/[σ^2^(*F*_o_^2^) + (0.0914*P*)^2^] where *P* = (*F*_o_^2^ + 2*F*_c_^2^)/3
3068 reflections	(Δ/σ)_max_ < 0.001
221 parameters	Δρ_max_ = 0.37 e Å^−^^3^
6 restraints	Δρ_min_ = −0.30 e Å^−^^3^

### Special details

**Table:**

Experimental. IR (KBr, cm^-1^): ν 3600–2000 (v br), 3543, 3427, 3340, 1622, 1585, 1525. Anal. Found: C, 54.86; H, 5.03; N, 14.07. Calculated for C_14_H_15_N_3_O_5_: C, 55.08; H, 4.95; N, 13.76%.
Geometry. All s.u.'s (except the s.u. in the dihedral angle between two l.s. planes) are estimated using the full covariance matrix. The cell s.u.'s are taken into account individually in the estimation of s.u.'s in distances, angles and torsion angles; correlations between s.u.'s in cell parameters are only used when they are defined by crystal symmetry. An approximate (isotropic) treatment of cell s.u.'s is used for estimating s.u.'s involving l.s. planes.
Refinement. Refinement of *F*^2^ against ALL reflections. The weighted *R*-factor *wR* and goodness of fit *S* are based on *F*^2^, conventional *R*-factors *R* are based on *F*, with *F* set to zero for negative *F*^2^. The threshold expression of *F*^2^ > 2σ(*F*^2^) is used only for calculating *R*-factors(gt) *etc*. and is not relevant to the choice of reflections for refinement. *R*-factors based on *F*^2^ are statistically about twice as large as those based on *F*, and *R*- factors based on ALL data will be even larger.

### Fractional atomic coordinates and isotropic or equivalent isotropic displacement parameters (Å^2^)

**Table:**

	*x*	*y*	*z*	*U*_iso_*/*U*_eq_	
O1	0.6688 (3)	−0.09682 (11)	0.3317 (3)	0.0350 (6)	
H1O	0.659 (5)	−0.0590 (7)	0.356 (5)	0.062 (13)*	
O2	1.1256 (3)	−0.07064 (10)	0.8502 (3)	0.0340 (6)	
H2O	1.189 (4)	−0.0952 (12)	0.921 (3)	0.034 (10)*	
O3	0.6421 (3)	0.18198 (10)	0.5589 (3)	0.0353 (6)	
O4	0.4717 (3)	0.25227 (10)	0.4047 (3)	0.0411 (7)	
N1	0.7166 (3)	0.00802 (11)	0.4628 (3)	0.0247 (6)	
N2	0.6577 (3)	0.06852 (12)	0.4550 (3)	0.0272 (6)	
H2N	0.702 (4)	0.0956 (11)	0.534 (3)	0.033 (9)*	
N3	0.5292 (3)	0.19801 (13)	0.4289 (3)	0.0315 (7)	
C1	0.8928 (4)	−0.07782 (14)	0.5935 (4)	0.0238 (7)	
C2	0.8060 (4)	−0.11805 (15)	0.4603 (4)	0.0288 (8)	
C3	0.8560 (4)	−0.17972 (15)	0.4541 (4)	0.0337 (8)	
H3	0.7969	−0.2048	0.3625	0.040*	
C4	0.9922 (4)	−0.20473 (15)	0.5816 (4)	0.0322 (8)	
H4	1.0262	−0.2473	0.5779	0.039*	
C5	1.0795 (4)	−0.16848 (14)	0.7142 (4)	0.0285 (8)	
H5	1.1721	−0.1863	0.8019	0.034*	
C6	1.0333 (4)	−0.10608 (14)	0.7204 (4)	0.0256 (7)	
C7	0.8350 (4)	−0.01181 (14)	0.5962 (3)	0.0228 (7)	
C8	0.9016 (4)	0.03103 (14)	0.7387 (4)	0.0314 (8)	
H8A	0.9969	0.0584	0.7212	0.043 (10)*	
H8B	0.9522	0.0063	0.8380	0.043 (9)*	
H8C	0.7996	0.0564	0.7506	0.067 (12)*	
C9	0.5300 (4)	0.09005 (14)	0.3180 (3)	0.0246 (7)	
C10	0.4639 (4)	0.15195 (14)	0.3020 (3)	0.0252 (7)	
C11	0.3303 (4)	0.17195 (15)	0.1615 (4)	0.0285 (8)	
H11	0.2881	0.2139	0.1538	0.034*	
C12	0.2596 (4)	0.13127 (15)	0.0345 (4)	0.0307 (8)	
H12	0.1681	0.1446	−0.0605	0.037*	
C13	0.3240 (4)	0.07063 (15)	0.0477 (4)	0.0313 (8)	
H13	0.2763	0.0424	−0.0399	0.038*	
C14	0.4557 (4)	0.05009 (15)	0.1848 (4)	0.0268 (7)	
H14	0.4973	0.0081	0.1895	0.032*	
O1W	0.6617 (3)	0.14329 (12)	0.9035 (3)	0.0397 (6)	
H1W	0.560 (3)	0.1348 (17)	0.836 (3)	0.060*	
H2W	0.714 (4)	0.1722 (13)	0.867 (4)	0.060*	

### Atomic displacement parameters (Å^2^)

**Table:**

	*U*^11^	*U*^22^	*U*^33^	*U*^12^	*U*^13^	*U*^23^
O1	0.0414 (14)	0.0315 (15)	0.0260 (13)	0.0007 (11)	0.0000 (10)	−0.0022 (11)
O2	0.0382 (14)	0.0312 (14)	0.0253 (13)	0.0020 (10)	−0.0021 (11)	−0.0004 (10)
O3	0.0420 (14)	0.0314 (14)	0.0275 (13)	−0.0038 (10)	0.0022 (11)	0.0001 (10)
O4	0.0468 (15)	0.0224 (14)	0.0475 (15)	0.0026 (11)	0.0032 (11)	−0.0025 (11)
N1	0.0256 (14)	0.0238 (15)	0.0257 (14)	−0.0001 (11)	0.0088 (11)	0.0013 (11)
N2	0.0336 (16)	0.0224 (16)	0.0228 (15)	−0.0007 (12)	0.0037 (12)	−0.0022 (12)
N3	0.0348 (17)	0.0299 (17)	0.0292 (16)	−0.0038 (13)	0.0083 (13)	0.0002 (13)
C1	0.0198 (16)	0.0295 (18)	0.0244 (16)	−0.0015 (13)	0.0099 (13)	0.0024 (13)
C2	0.0312 (18)	0.030 (2)	0.0240 (17)	−0.0031 (14)	0.0069 (14)	0.0007 (14)
C3	0.043 (2)	0.029 (2)	0.0288 (18)	−0.0028 (15)	0.0103 (16)	−0.0062 (15)
C4	0.0382 (19)	0.0231 (19)	0.038 (2)	−0.0017 (15)	0.0150 (16)	−0.0007 (15)
C5	0.0317 (18)	0.0274 (19)	0.0275 (18)	0.0004 (14)	0.0103 (14)	0.0044 (14)
C6	0.0298 (18)	0.0255 (19)	0.0225 (16)	−0.0049 (14)	0.0092 (14)	−0.0029 (13)
C7	0.0193 (16)	0.0277 (19)	0.0215 (16)	−0.0010 (13)	0.0062 (13)	0.0021 (13)
C8	0.036 (2)	0.0264 (19)	0.0254 (18)	0.0007 (15)	−0.0015 (15)	−0.0014 (14)
C9	0.0279 (18)	0.0288 (19)	0.0168 (16)	−0.0020 (14)	0.0059 (13)	0.0003 (13)
C10	0.0294 (17)	0.0246 (19)	0.0226 (17)	−0.0046 (14)	0.0092 (14)	−0.0027 (13)
C11	0.0307 (18)	0.0254 (18)	0.0315 (18)	−0.0007 (14)	0.0124 (14)	0.0042 (14)
C12	0.0272 (18)	0.036 (2)	0.0265 (18)	0.0030 (15)	0.0040 (14)	0.0035 (15)
C13	0.0338 (19)	0.035 (2)	0.0239 (18)	0.0005 (15)	0.0061 (14)	−0.0035 (14)
C14	0.0263 (17)	0.0276 (18)	0.0253 (17)	0.0010 (14)	0.0059 (13)	−0.0004 (14)
O1W	0.0406 (15)	0.0385 (16)	0.0343 (14)	−0.0005 (12)	0.0019 (11)	0.0024 (12)

### Geometric parameters (Å, º)

**Table:**

O1—C2	1.359 (4)	C5—C6	1.387 (4)
O1—H1O	0.844 (10)	C5—H5	0.9500
O2—C6	1.361 (3)	C7—C8	1.494 (4)
O2—H2O	0.844 (10)	C8—H8A	0.9800
O3—N3	1.242 (3)	C8—H8B	0.9800
O4—N3	1.238 (3)	C8—H8C	0.9800
N1—C7	1.305 (3)	C9—C14	1.407 (4)
N1—N2	1.366 (3)	C9—C10	1.411 (4)
N2—C9	1.370 (4)	C10—C11	1.399 (4)
N2—H2N	0.880 (10)	C11—C12	1.376 (4)
N3—C10	1.445 (4)	C11—H11	0.9500
C1—C6	1.422 (4)	C12—C13	1.381 (4)
C1—C2	1.430 (4)	C12—H12	0.9500
C1—C7	1.483 (4)	C13—C14	1.378 (4)
C2—C3	1.380 (4)	C13—H13	0.9500
C3—C4	1.379 (4)	C14—H14	0.9500
C3—H3	0.9500	O1W—H1W	0.841 (10)
C4—C5	1.377 (4)	O1W—H2W	0.845 (10)
C4—H4	0.9500		
			
C2—O1—H1O	103 (3)	N1—C7—C8	120.1 (3)
C6—O2—H2O	107 (2)	C1—C7—C8	124.5 (2)
C7—N1—N2	119.1 (3)	C7—C8—H8A	109.5
N1—N2—C9	120.2 (2)	C7—C8—H8B	109.5
N1—N2—H2N	123 (2)	H8A—C8—H8B	109.5
C9—N2—H2N	117 (2)	C7—C8—H8C	109.5
O4—N3—O3	122.0 (3)	H8A—C8—H8C	109.5
O4—N3—C10	119.1 (3)	H8B—C8—H8C	109.5
O3—N3—C10	119.0 (3)	N2—C9—C14	120.6 (3)
C6—C1—C2	115.2 (3)	N2—C9—C10	123.0 (3)
C6—C1—C7	123.8 (3)	C14—C9—C10	116.3 (3)
C2—C1—C7	121.0 (3)	C11—C10—C9	121.5 (3)
O1—C2—C3	116.5 (3)	C11—C10—N3	116.4 (3)
O1—C2—C1	121.0 (3)	C9—C10—N3	122.2 (3)
C3—C2—C1	122.5 (3)	C12—C11—C10	120.5 (3)
C4—C3—C2	119.7 (3)	C12—C11—H11	119.8
C4—C3—H3	120.2	C10—C11—H11	119.8
C2—C3—H3	120.2	C11—C12—C13	118.8 (3)
C3—C4—C5	120.5 (3)	C11—C12—H12	120.6
C3—C4—H4	119.8	C13—C12—H12	120.6
C5—C4—H4	119.8	C14—C13—C12	121.6 (3)
C4—C5—C6	120.5 (3)	C14—C13—H13	119.2
C4—C5—H5	119.8	C12—C13—H13	119.2
C6—C5—H5	119.8	C13—C14—C9	121.3 (3)
O2—C6—C5	119.4 (3)	C13—C14—H14	119.3
O2—C6—C1	118.9 (3)	C9—C14—H14	119.3
C5—C6—C1	121.6 (3)	H1W—O1W—H2W	111 (3)
N1—C7—C1	115.5 (3)		
			
C7—N1—N2—C9	−178.3 (3)	C6—C1—C7—C8	−7.0 (5)
C6—C1—C2—O1	179.6 (3)	C2—C1—C7—C8	172.1 (3)
C7—C1—C2—O1	0.5 (4)	N1—N2—C9—C14	0.9 (4)
C6—C1—C2—C3	−0.7 (4)	N1—N2—C9—C10	−179.8 (3)
C7—C1—C2—C3	−179.8 (3)	N2—C9—C10—C11	−178.6 (3)
O1—C2—C3—C4	−179.0 (3)	C14—C9—C10—C11	0.7 (4)
C1—C2—C3—C4	1.3 (5)	N2—C9—C10—N3	1.2 (5)
C2—C3—C4—C5	−0.5 (5)	C14—C9—C10—N3	−179.5 (3)
C3—C4—C5—C6	−0.8 (5)	O4—N3—C10—C11	−3.3 (4)
C4—C5—C6—O2	−177.8 (3)	O3—N3—C10—C11	177.0 (3)
C4—C5—C6—C1	1.5 (5)	O4—N3—C10—C9	176.9 (3)
C2—C1—C6—O2	178.6 (3)	O3—N3—C10—C9	−2.8 (4)
C7—C1—C6—O2	−2.3 (4)	C9—C10—C11—C12	0.0 (4)
C2—C1—C6—C5	−0.7 (4)	N3—C10—C11—C12	−179.8 (3)
C7—C1—C6—C5	178.4 (3)	C10—C11—C12—C13	−0.6 (5)
N2—N1—C7—C1	−179.4 (2)	C11—C12—C13—C14	0.4 (5)
N2—N1—C7—C8	1.2 (4)	C12—C13—C14—C9	0.3 (5)
C6—C1—C7—N1	173.6 (3)	N2—C9—C14—C13	178.5 (3)
C2—C1—C7—N1	−7.4 (4)	C10—C9—C14—C13	−0.8 (4)

### Hydrogen-bond geometry (Å, º)

**Table:**

*D*—H···*A*	*D*—H	H···*A*	*D*···*A*	*D*—H···*A*
O1—H1*O*···N1	0.85 (2)	1.69 (2)	2.489 (3)	157 (4)
N2—H2*N*···O3	0.88 (2)	1.93 (2)	2.602 (3)	132 (2)
O2—H2*O*···O1*W*^i^	0.84 (3)	1.90 (3)	2.742 (3)	174 (2)
O1*W*—H1*W*···O1^ii^	0.84 (2)	2.08 (3)	2.910 (3)	169 (3)
O1*W*—H2*W*···O4^iii^	0.85 (3)	2.50 (3)	3.256 (3)	150 (3)
C11—H11···O3^iv^	0.95	2.52	3.447 (4)	166

Symmetry codes: (i) −*x*+2, −*y*, −*z*+2; (ii) −*x*+1, −*y*, −*z*+1; (iii) *x*+1/2, −*y*+1/2, *z*+1/2; (iv) *x*−1/2, −*y*+1/2, *z*−1/2.
